# Protective effect of resveratrol and quercetin on in vitro-induced diabetic mouse corpus cavernosum

**DOI:** 10.1186/s12933-016-0366-9

**Published:** 2016-03-18

**Authors:** Charlotte Boydens, Bart Pauwels, Laura Vanden Daele, Johan Van de Voorde

**Affiliations:** Department of Pharmacology, Ghent University, De Pintelaan 185, 9000 Ghent, Belgium

**Keywords:** Resveratrol, Quercetin, Diabetes, Erectile dysfunction

## Abstract

**Background:**

Hyperglycemia and increased levels of methylglyoxal (MGO) can trigger the development of vascular complications in diabetes. Resveratrol and quercetin are red wine polyphenols with known beneficial cardiovascular properties, including an antioxidant capacity. This study evaluated whether resveratrol and/or quercetin could prevent in vitro-induced diabetic changes in neurogenic and vascular relaxant responses of mouse arteries and corpora cavernosa.

**Methods:**

Isometric tension of isolated aorta, mesenteric arteries and corpora cavernosa was measured using organ bath systems. Diabetic conditions were mimicked in vitro by co-incubating the tissues for 2 h with high glucose (HG, 30 mM) and MGO (120 µM).

**Results:**

The presence of HG and MGO significantly blunted acetylcholine (Ach)-induced relaxations in corpora cavernosa and mesenteric arteries but not in aorta. Electrical field stimulated (EFS) responses of corpora cavernosa were also significantly inhibited by these diabetic conditions. In corpora cavernosa 2 h co-incubation with resveratrol (30 µM) or quercetin (30 µM) significantly attenuated HG and MGO-induced deficits in Ach- and EFS-responses.

**Conclusions:**

Our study demonstrates that in mouse arteries, HG and MGO rather affect endothelium derived hyperpolarizing factor-mediated than nitric oxide (NO)-mediated relaxations. In corpora cavernosa HG and MGO interfere with NO release. Resveratrol and quercetin protect mouse corpora cavernosa from diabetic-induced damage to NO-mediated relaxant responses. This might rely on their antioxidant capacity.

## Background

Hyperglycemia and increased levels of methylglyoxal (MGO), a reactive metabolite produced during glycolysis, are two hallmarks of diabetes mellitus which can trigger the development of vascular complications in diabetes [1–5]. These vascular events are presumed to underlie the pathogenesis of diabetic-related erectile dysfunction (ED). Indeed, in different models of diabetes it has been demonstrated that neurogenic- and endothelium-mediated relaxation of isolated corpora cavernosa [6–10] or arteries [5, 11, 12] is impaired. Moreover, in vitro research showed that acute exposure of isolated arteries to high glucose (HG) or MGO affects endothelium-dependent relaxations in different vascular beds [1, 3, 13–15]. Oxidative stress may play a causative role in the damaging effect of hyperglycemia or MGO on the endothelium [1, 3, 5, 14, 15]. Therefore, antioxidant therapy might offer an option for treating diabetic complications, including ED. Resveratrol and quercetin are two naturally occurring polyphenols, mainly present in the skin of grapes and thus abundant in red wine. Both polyphenols are suggested to have beneficial cardiovascular properties, including a relaxant [16, 17] and antioxidant [16, 18, 19] capacity. Recently, our group reported a relaxant and antioxidant effect of resveratrol in isolated mouse corpora cavernosa [20]. Considering their antioxidant properties resveratrol and/or quercetin could potentially be of value in the treatment of diabetic ED. However, the antioxidant effect of these polyphenols in mouse mesenteric arteries and corpora cavernosa in diabetic conditions is as yet unexplored. Therefore, the aim of this study was to evaluate whether resveratrol and/or quercetin could protect mouse mesenteric artery and/or corpora cavernosa from HG and MGO-induced defects in endothelium- and neurogenic-mediated relaxations.

## Methods

### Animals

Adult (8–12 weeks) male Swiss mice were obtained from Janvier (Saint-Berthevin, France). Food and water was provided ad libitum and all animals were treated in accordance with the Guide for the Care and Use of Laboratory Animals published by the US National Institutes of Health. This study was approved by the local Ethical Committee for Animal experiments, Faculty of Medicine and Health Sciences, Ghent University, Belgium.

### Tissue preparation

After cervical dislocation, thoracic aorta, mesenteric arteries (1st and 2nd order) and corpora cavernosa were carefully isolated and dissected free from surrounding structures. All tissues were kept in cold Krebs–Ringer bicarbonate (KRB) solution. Aorta and mesenteric arteries were cut into rings of about 3 mm in length and mounted in a wire myograph for isometric tension measurements. Of each mouse, one corpora cavernosa was mounted horizontally in a myograph with one end fixed to a transducer and the other to a micrometer. The tissue chambers were filled with 10 mL KRB solution at 37 °C (pH 7.4) equilibrated with 95 % O_2_–5 % CO_2_. The preparations were allowed to equilibrate for 30 min in KRB solution that was frequently replaced. In order to obtain maximal, stable contractions and relaxations, mesenteric arteries were stretched to their optimal lumen diameter that gives a maximum response, as calculated on the basis of the passive wall tension-internal circumference relationship [21]. Aorta segments and corpora cavernosa were gradually stretched until a stable preload of 0.5 g (aorta) or 0.45 g (corpora cavernosa) was obtained. Subsequently, tissues were repeatedly activated using different protocols: (1) aorta with 120 mM K^+^ and 1 µM phenylephrine (Phe) (2) mesenteric arteries three times with 120 mM K^+^ and 10 µM Phe and (3) corpora cavernosa two times with 5 µM Phe. The integrity of the endothelium was examined by contracting the tissues with submaximal Phe concentrations (1 µM for aorta, 5 µM for corpora cavernosa and 10 µM for mesenteric arteries) and adding acetylcholine (Ach) (10 µM for arteries or 1 µM for corpora cavernosa). Only tissues that relaxed more than 50 % to Ach, were included in this study.

### Experimental protocol

Tissues were incubated for 2 h with: (1) control KRB solution containing 10 mM glucose or (2) KRB solution containing 30 mM glucose (HG) and 120 µM MGO. Every 30 min the KRB solution in the organ baths was refreshed. After 2 h incubation, tissues were precontracted with Phe (1–10 µM). Firstly, the relaxant effect of resveratrol (1–100 µM) and quercetin (1–100 µM) was evaluated. Secondly, the protective effect of resveratrol and quercetin on endothelium-(in) dependent relaxations were evaluated. Relaxant responses to Ach (1 nM to 1 or 10 µM) and sodium nitroprusside (SNP) (1 nM–1 µM), were examined after 2 h pretreatment with 30 mM glucose and 120 µM MGO in absence or presence of ascorbic acid (100 µM), tempol (100 µM), resveratrol (30 µM) or quercetin (10 or 30 µM). In corpora cavernosa, neurogenic-mediated relaxations were also studied by applying electrical field stimulation (EFS; parameters: train duration 40 s; 1, 2, 4 and 8 Hz; pulse duration 5 min and 80 V).

### Drugs and chemicals

The experiments were performed in a KRB solution of the following composition (mM): NaCl 135, KCl 5, NaHCO_3_ 20, glucose 10 (control) or 30 (HG), CaCl_2_ 2.5, MgSO_4_ 1.3, KH_2_PO_4_ 1.2 and EDTA 0.026 in H_2_O. l-phenylephrine hydrochloride (Phe), acetylcholine chloride (Ach), SNP, Nω-Nitro-l-arginine methyl ester hydrochloride (L-NAME), indomethacin (indo), MGO solution, 4-hydroxy-tempo (Temp), l-ascorbic acid (AA), resveratrol, quercetin and dimethylsulfoxide (DMSO) were obtained from Sigma (St. Louis, MO). Stock solutions were made in water except for indo (ethanol). The final concentrations of vehicle solution in the organ bath never exceeded 0.1 %. Stock solutions of 100 mM of resveratrol and quercetin were made in DMSO, but were further diluted in distilled water (10 mM) before adding to the organ baths.

### Statistics

The data are presented as mean values ± SEM. Relaxations are expressed as % decrease in precontractile tone. N is the number of individual strips/rings used. Sensitivity (pD2) and maximum response (E_max_) were calculated from the concentration–response curves to resveratrol, quercetin or acetylcholine. pD2 was defined as the negative logarithm to base 10 of the EC50 values and E_max_ was defined as the maximal relaxation. The data were analysed using SPSS, version 22; IBM Corporation, Armonk, NY, USA. Statistical significance was evaluated using the Mann–Whitney U test. Two groups of data were considered significantly different when p < 0.05. Graphs were created using Graphpad Prism, version 4.00, GraphPad Software, San Diego California USA.

## Results

### Relaxant capacity of resveratrol and quercetin in presence of HG and MGO

**Fig. 1:**
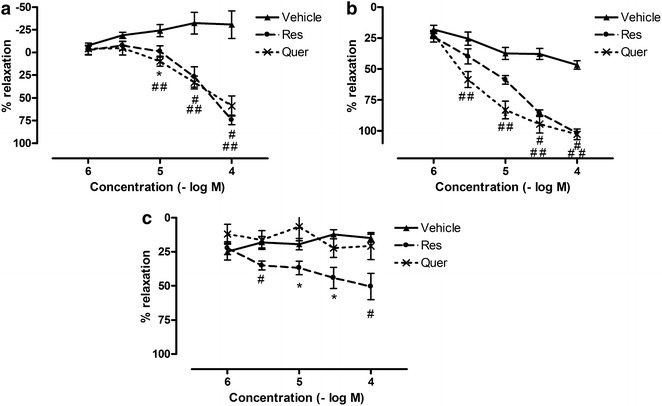
(Vaso)relaxant effect of resveratrol or quercetin in presence of high glucose and methylglyoxal. **a** Aorta, **b** mesenteric artery and **c** corpora cavernosa. Tissues were incubated for 2 h with 30 mM glucose (HG) and 120 µM methylglyoxal (MGO) before constructing concentration–response (1–100 µM) curves to resveratrol (Res, 15 min) or quercetin (Quer, 15 min). Data are expressed as % decrease of Phe-induced tone; Mann–Whitney U test; ^#^p < 0.01 and *p < 0.05 (vehicle control vs. Res) (n = 5–6); ^##^p < 0.01 and **p < 0.05 (vehicle control vs. Quer) (n = 5–6)

Both resveratrol and quercetin (1–100 µM, 15 min) relaxed Phe-precontracted aortic and mesenteric rings concentration dependently (Fig. 1a, b). Maximal relaxation for resveratrol was 74.4 ± 5.1 % (aorta) and 101.8 ± 2.0 % (mesenteric arteries); pD2 values were 4.4 ± 0.1 and 4.9 ± 0.1 for aortic and mesenteric rings respectively. Maximal relaxation for quercetin was 59.0 ± 11.1 % (aorta) and 102.8 ± 4.3 % (mesenteric arteries); pD2 values were 4.5 ± 0.1 and 5.4 ± 0.1 for aortic and mesenteric rings respectively. In contrast, only resveratrol evoked concentration dependent relaxations in corpora cavernosa (E_max_: 50.6 ± 9.6 % and pD2: 4.9 ± 0.5) (Fig. 1c). Based on these results, following concentrations of resveratrol and quercetin were chosen for our subsequent experiments: 10 µM resveratrol and 3 µM quercetin for mesenteric arteries, 30 µM resveratrol and 30 µM quercetin for corpora cavernosa.

### HG and MGO impair Ach- and EFS-relaxations

Endothelium-dependent relaxations where evaluated using Ach (1 nM to 1 or 10 µM), which induced concentration dependent relaxations in all tissues. To explore the role of endothelium derived hyperpolarizing factor (EDHF), Ach-curves were constructed in presence of the NO synthase inhibitor, L-NAME (0.1 mM, 20 min), and the cyclooxygenase blocker, indo (10 µM, 20 min). The presence of L-NAME and indo completely abolished Ach-relaxations in aorta and corpora cavernosa, whereas these inhibitors did not affect Ach-relaxations of mesenteric arteries (data not shown).

**Fig. 2:**
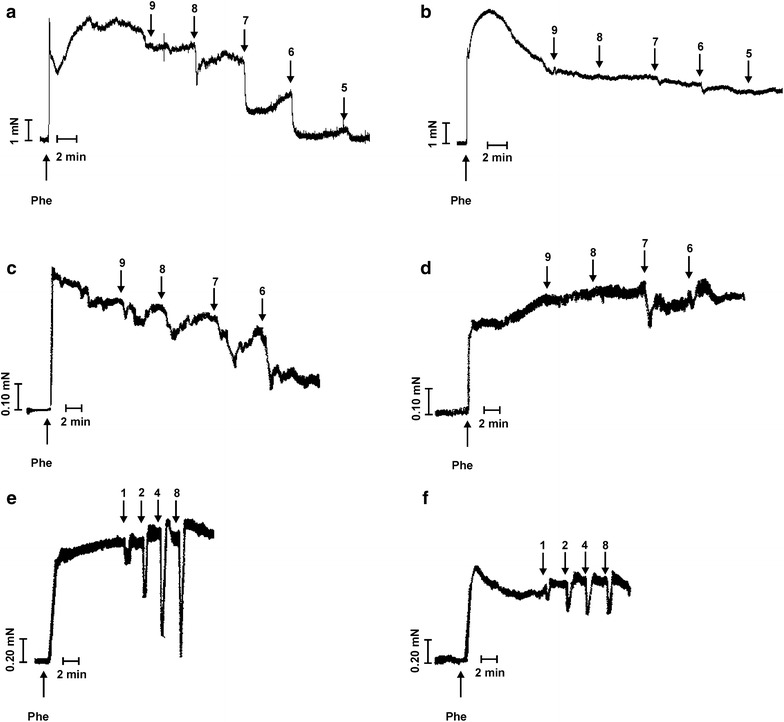
Effect of in vitro-diabetic conditions on acetylcholine and electrical field stimulated responses of mesenteric artery and corpora cavernosa. Original tracings showing the effect of 2 h incubation with high glucose (HG, 30 mM) and methylglyoxal (MGO, 120 µM) on (**a**–**d**) acetylcholine (Ach, −log M) and **e**, **f** electrical field stimulated (Hz) relaxations of mesenteric artery and corpora cavernosa after precontraction with phenylephrine (Phe; 10 µM for mesenteric arteries; 5 µM for corpora cavernosa). Response curve to Ach in mesenteric artery in (**a**) absence and (**b**) presence of HG and MGO; in corpora cavernosa in (**c**) absence and (**d**) presence of HG and MGO. EFS-relaxations in corpora cavernosa in (**e**) absence and (**f**) presence of HG and MGO

After 2 h incubation with HG (30 mM) and MGO (120 µM), Ach-induced relaxations were significantly blunted in mesenteric arteries (Fig. 2a, b) and corpora cavernosa (Fig. 2c, d). EFS-relaxations of corpora cavernosa were significantly reduced by 2 h exposure to HG (30 mM) and MGO (120 µM) (Fig. 2e, f).

**Fig. 3:**
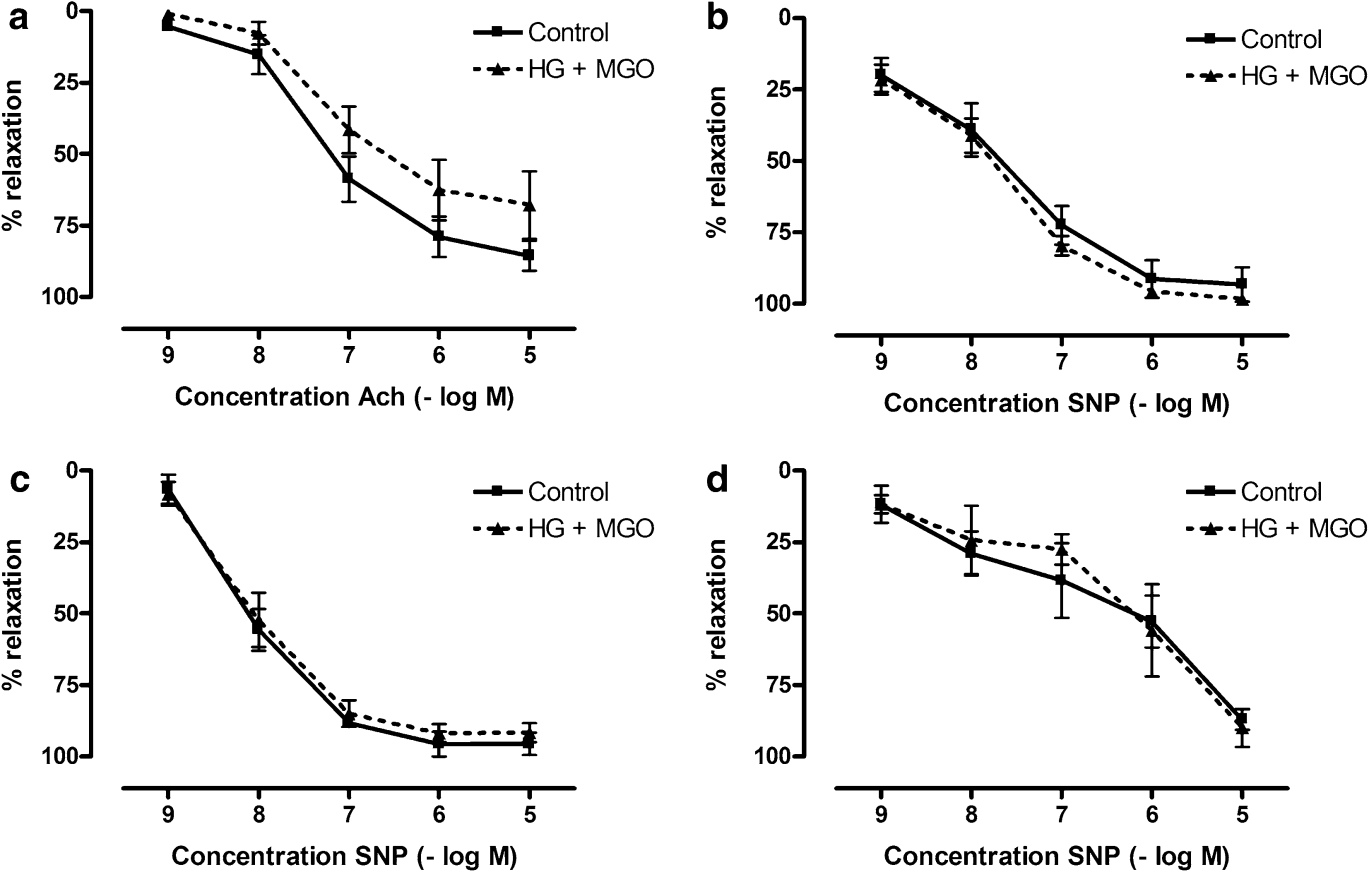
Effect of in vitro-diabetic conditions on acetylcholine and sodium nitroprusside responses of aorta, mesenteric artery and corpora cavernosa. Acetylcholine (Ach)-induced relaxations of mouse (**a**) aorta and on sodium nitroprusside (SNP)-induced relaxations of (**b**) aorta, **c** mesenteric artery and **d** corpora cavernosa after 2 h incubation with high glucose (HG, 30 mM) and methylglyoxal (MGO, 120 µM) after precontraction with Phe (1 µM for aorta, 10 µM for mesenteric arteries and 5 µM for corpora cavernosa). Data are expressed as % decrease of Phe-induced tone; Mann–Whitney U test; (n = 7–8 for Ach-curves) (n = 4–6 for SNP-curves)

In contrast, the presence of 30 mM glucose and 120 µM MGO neither affected Ach-relaxations of aorta (Fig. 3a) nor SNP-relaxations of aorta, mesenteric arteries or corpora cavernosa (Fig. 3b–d).

### Role of oxidative stress in glucose and MGO-impaired relaxations

**Fig. 4:**
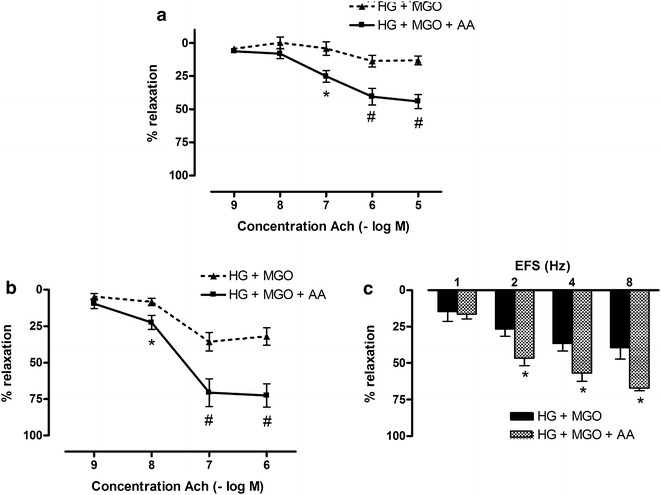
Ascorbic acid prevents high glucose and methylglyoxal-induced deficits in mesenteric artery and corpora cavernosa. Acetylcholine (Ach)-induced relaxations of **a** mesenteric artery and **b** corpora cavernosa after 2 h co-incubation with high glucose (HG, 30 mM) and methylglyoxal (MGO, 120 µM) in absence and presence of ascorbic acid (AA, 100 µM) after precontraction with Phe (10 µM for mesenteric arteries; 5 µM for corpora cavernosa). **c** Electrical field stimulated (EFS) relaxations of corpora cavernosa after 2 h incubation with high glucose (HG, 30 mM) and methylglyoxal (MGO, 120 µM) in absence and presence of ascorbic acid (AA, 100 µM). Data are expressed as % decrease of Phe-induced tone; Mann–Whitney U test; ^#^p < 0.01 and *p < 0.05 (n = 5–7)

**Fig. 5:**
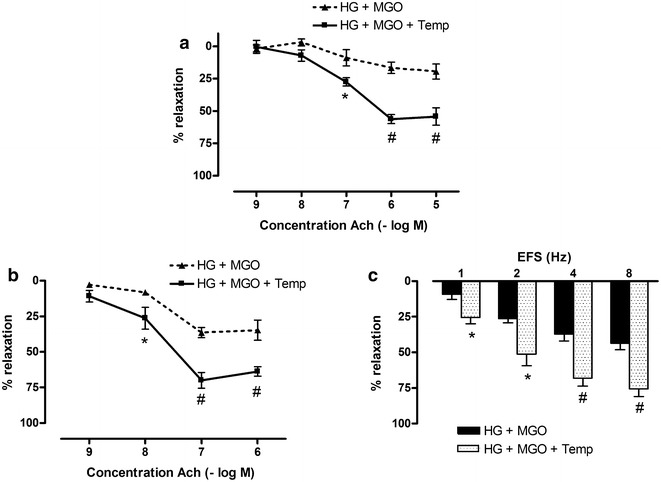
Tempol prevents high glucose and methylglyoxal-induced deficits in mesenteric artery and corpora cavernosa. Acetylcholine (Ach)-induced relaxations of **a** mesenteric artery and **b** corpora cavernosa after 2 h incubation with high glucose (HG, 30 mM) and methylglyoxal (MGO, 120 µM) in absence and presence of tempol (Temp, 100 µM) after precontraction with Phe (10 µM for mesenteric arteries; 5 µM for corpora cavernosa). **c** Electrical field stimulated (EFS) relaxations of 5 µM Phe-precontracted mouse corpora cavernosa after 2 h incubation with high glucose (HG, 30 mM) and methylglyoxal (MGO, 120 µM) in absence and presence of tempol (Temp, 100 µM). Data are expressed as % decrease of Phe-induced tone; Mann–Whitney U test; ^#^p < 0.01 and *p < 0.05 (n = 5–6)

Mesenteric arteries and corpora cavernosa were pretreated for 2 h with 30 mM glucose and 120 µM MGO in combination with the antioxidant AA (100 µM) or the superoxide anion scavenger Temp (100 µM). In mesenteric arteries both AA (Fig. 4a) and Temp (Fig. 5a) prevented glucose and MGO impairment of Ach-relaxations. Neither AA nor Temp altered sensitivity to Ach, whereas maximal Ach-responses were improved in presence of AA or Temp (Table 1). Similarly, in corpora cavernosa, both antioxidants counteracted the effect of glucose and MGO incubation on Ach- and EFS-responses (Figs. 4b, c and 5b, c). pD2 values were unaltered by antioxidant treatment. Maximal Ach-responses were however improved in presence of AA or Temp (Table 1).

**Table 1:** Effect of antioxidants and polyphenol treatment on acetylcholine-relaxations of mesenteric arteries and corpora cavernosa

	Mesenteric arteries		Corpora cavernosa	
	pD2	E_max_ (%)	pD2	E_max_ (%)
HG + MGO HG + MGO + AA	7.1 ± 0.4 7.0 ± 0.2	19.8 ± 2.6 46.8 ± 4.8^#^	7.8 ± 0.1 7.8 ± 0.2	35.8 ± 6.4 83.5 ± 6.1*
HG + MGO HG + MGO + Temp	7.0 ± 0.3 7.1 ± 0.1	23.9 ± 5.04 58.3 ± 4.4^#^	7.7 ± 0.1 7.9 ± 0.2	38.7 ± 4.2 74.3 ± 3.5^#^
HG + MGO HG + MGO + Res	6.3 ± 0.9 6.7 ± 0.5	23.2 ± 5.0 34.8 ± 3.7	7.7 ± 0.3 7.6 ± 0.2	47.6 ± 4.3 83.8 ± 4.6^#^
HG + MGO HG + MGO + Quer	7.4 ± 1.0 6.7 ± 0.2	24.4 ± 4.4 31.3 ± 6.3	7.9 ± 0.3 7.9 ± 0.02	44.9 ± 3.5 80.0 ± 5.5^#^

*pD2* −log(EC_50_), *E*_*max*_ maximal relaxation response, *HG* high glucose (30 mM), *MGO* methylglyoxal (120 µM), *AA* ascorbic acid (100 µM), *Temp* tempol (100 µM), *Res* resveratrol (10 µM for mesenteric arteries, 30 µM for corpora cavernosa), *Quer* quercetin (3 µM for mesenteric arteries; 30 µM for corpora cavernosa). Data are mean ± SEM. * p < 0.05; ^#^p < 0.01 (compared to HG + MGO group)

### Effect of resveratrol and quercetin on glucose and MGO-impaired relaxations

**Fig. 6:**
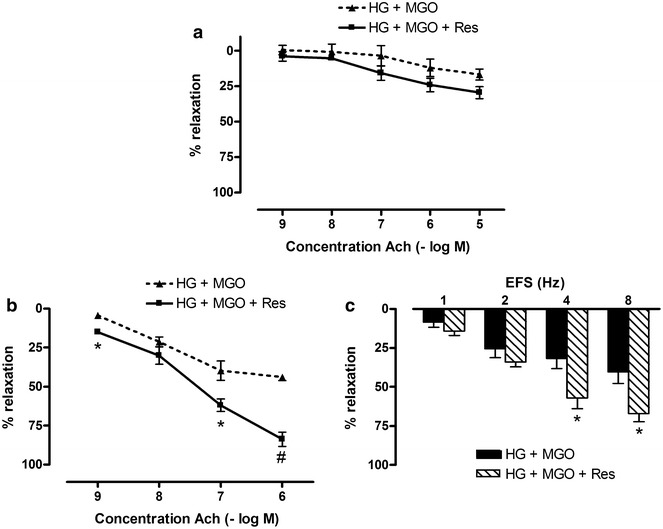
Effect of resveratrol on high glucose and methylglyoxal-induced deficits in mesenteric artery and corpora cavernosa. Acetylcholine (Ach)-induced relaxations of **a** mesenteric artery and **b** corpora cavernosa after 2 h incubation with high glucose (HG, 30 mM) and methylglyoxal (MGO, 120 µM) in absence and presence of resveratrol (Res, 10 µM for **a**; 30 µM for **b**) after precontraction with Phe (10 µM for mesenteric arteries; 5 µM for corpora cavernosa). **c** Electrical field stimulated (EFS) relaxations of 5 µM Phe-precontracted mouse corpora cavernosa after 2 h incubation with high glucose (HG, 30 mM) and methylglyoxal (MGO, 120 µM) in absence and presence of resveratrol (Res, 30 µM). Data are expressed as % decrease of Phe-induced tone; Mann–Whitney U test; ^#^p < 0.01 and *p < 0.05 (n = 5–7)

**Fig. 7:**
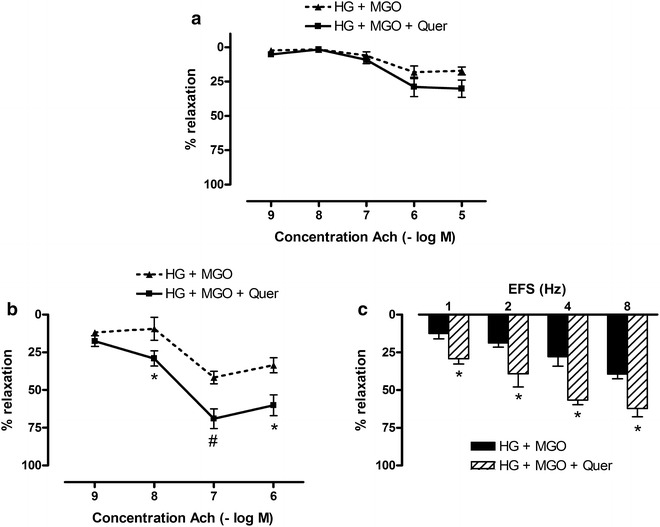
Effect of quercetin on high glucose and methylglyoxal-induced deficits in mesenteric artery and corpora cavernosa. Acetylcholine (Ach)-induced relaxations of **a** mesenteric artery and **b** corpora cavernosa after 2 h incubation with high glucose (HG, 30 mM) and methylglyoxal (MGO, 120 µM) in absence and presence of quercetin (Quer, 3 µM for **a**; 30 µM for **b**) after precontraction with Phe (10 µM for mesenteric arteries; 5 µM for corpora cavernosa). **c** Electrical field stimulated (EFS) relaxations of 5 µM Phe-precontracted mouse corpora cavernosa after 2 h incubation with high glucose (HG, 30 mM) and methylglyoxal (MGO, 120 µM) in absence and presence of quercetin (Quer, 30 µM). Data are expressed as % decrease of Phe-induced tone; Mann–Whitney U test; ^#^p < 0.01 and *p < 0.05 (n = 4–7)

In mesenteric arteries, Ach-induced relaxations were explored after 2 h pretreatment with 30 mM glucose and 120 µM MGO in combination with resveratrol (Res, 10 µM, 2 h) or quercetin (Quer, 3 µM, 2 h). Neither resveratrol (Fig. 6a) nor quercetin (Fig. 7a) prevented glucose and MGO impairment of Ach-relaxations as neither E_max_ nor pD2 values were affected by resveratrol or quercetin treatment (Table 1). On the contrary, exposing the corpora cavernosa for 2 h to 30 mM glucose and 120 µM MGO in combination with resveratrol (30 µM, 2 h) or quercetin (30 µM, 2 h) significantly corrected blunted Ach- and EFS-relaxations (Figs. 6b, c and 7b, c). E_max_ values were significantly corrected by resveratrol as well as by quercetin treatment (Table 1). No effect of both polyphenols was found on the potency of Ach as pD2 values were unchanged by resveratrol or quercetin addition (Table 1).

## Discussion

The present study was the first to demonstrate that co-incubation of mouse mesenteric arteries and corpora cavernosa with 30 mM glucose and 120 µM MGO causes deficits in endothelium- and neurogenic-mediated relaxations. In addition, resveratrol and quercetin were effective in preventing diabetic-induced deficits in corpora cavernosa.

### Diabetic (neuro)vascular damage induced by in vitro HG and MGO treatment

The concentrations of glucose and MGO used in our study were chosen based on literature. 30 mM glucose is a level that is found or even exceeded in the blood of db/db mouse model of type 2 diabetes that is associated with severe endothelial dysfunction in the small mesenteric arteries [22]. In addition, in patients with poorly controlled diabetes, the MGO level in blood can raise up to 400 µM [23]. Moreover, exogenous MGO is not fully absorbed intracellularly [24]. Therefore the use of 30 mM glucose and 120 µM MGO in this study seems to be justified as these concentrations seem pathophysiologically relevant.

The effect of either glucose or MGO on endothelium-dependent relaxations has been investigated in several studies [1, 3, 5, 13, 15], however no study has evaluated the joint effect of MGO and elevated glucose in mouse mesenteric arteries or corpora cavernosa. Since both glucose and MGO levels are increased in diabetes mellitus [4, 23], diabetic conditions are more precisely mimicked in vitro by co-incubating the tissues with both compounds. Our results are in line with previous studies showing a negative effect of HG or MGO on endothelium-dependent relaxations in mesenteric arteries of rats or mice [1, 13–15]. In addition, the impaired endothelium- and neurogenic-mediated relaxations observed in corpora cavernosa are in agreement with studies using other animal models for diabetes [6–10]. It thus seems that the in vitro glucose/MGO treatment used in this study indeed yields neurovascular damage as observed in vivo in diabetes mellitus. Confirming most of these studies, relaxant responses to SNP, an endothelium-independent nitric oxide (NO) donor, were unaltered by exposing the tissues to HG and MGO. Thus neither a diminished response at the level of the smooth muscle cells nor defects in the cyclic guanosine monophosphate pathway can explain the observed diabetic-induced deficits of Ach- and EFS-relaxations. Instead, these deficits will thus rather result from decreased synthesis and/or increased quenching of endothelium- or neuronal-derived relaxing factors by HG and MGO.

### Aorta versus mesenteric arteries

No effect of diabetic conditions was found on Ach-relaxations in aorta, which is in contrast to Dhar et al. [3], who found a decreased Ach response in rat aorta after incubation with 25 mM glucose. As in our study a trend in reduced Ach-relaxations of aorta could be observed, it is possible that the incubation time of glucose/MGO was too short to cause endothelial damage. With respect to the arteries used in this study, our results indicate that diabetic conditions affect endothelium-mediated relaxations in mesenteric arteries more rapidly than in aorta. This implies that the type of artery and/or the lumen diameter determines the effect of glucose/MGO on Ach-induced relaxations. Ach stimulates the release of endothelium derived relaxing factors: NO, prostacyclin and EDHF. Different vascular beds exhibit a marked heterogeneity in the relative contribution of these factors to endothelium-dependent relaxations [25]. Indeed, inhibition of NO and prostacyclin production (using L-NAME and indo respectively) did not affect Ach-relaxations in mesenteric arteries whereas the presence of these inhibitors completely abolished Ach-responses in aorta. This demonstrates that Ach-relaxations in mesenteric arteries are EDHF-mediated [11, 25] while in aorta these are NO-mediated. Thus, in mouse arteries HG and MGO seem to interfere more rapidly with EDHF- than with NO-dependent relaxations.

### Corpora cavernosa

Like in aorta, Ach- and EFS-relaxations of corpora cavernosa are NO-mediated (own observations and [9, 26]). However, unlike in aorta, these NO-mediated relaxations are inhibited by co-incubating the corpora cavernosa with 30 mM glucose and 120 µM MGO. So, besides the lumen diameter of the vessels, the type of tissue also contributes to the effect of diabetic conditions on relaxant responses to Ach or EFS. Since unaltered aortic Ach-relaxations were observed in presence of HG and MGO, it can be assumed that (1) these diabetic conditions induce less damage in aorta or (2) the aorta is more resistant to changes evoked by glucose/MGO. As previously mentioned, our results cannot rule out that longer exposure to HG/MGO could yield similar endothelial damage in aorta as seen in corpora cavernosa or mesenteric arteries. However, based on our results it seems that both corpora cavernosa and mesenteric arteries are more prone to diabetic-induced endothelial and/or neurogenic changes compared to large arteries such as aorta.

### Oxidative stress as underlying cause of in vitro-diabetic damage

A hyperglycemia or MGO-dependent increase in reactive oxygen species (ROS) [1, 3, 5, 14, 15] seems to be an important contributor to the pathogenesis of the diabetic-(neuro)vascular complications. After all, antioxidant administration can limit oxidative damage and restore diabetes-induced defects in endothelial- and/or neurogenic responses of arteries [27–29] or corpora cavernosa [7–10]. Here we demonstrate that the antioxidant AA or the superoxide anion scavenger Temp markedly inhibit glucose/MGO-induced damage to endothelium- and neurogenic-mediated responses of mesenteric arteries and corpora cavernosa. Despite the large amount of evidence for HG or MGO-induced oxidative stress, the mechanisms whereby ROS formation is induced, are not fully understood. Recently it was reported that MGO-stimulated ROS production activates the endothelial plasma membrane transport protein, Na^+^/H^+^ exchanger (NHE1) [30]. Moreover HG-induced vascular deficits are related to hyperactivity of NHE1 [31]. Activation of NHE1 may thus be a critical step for ROS-induced damage. Regardless of the mechanism by which glucose/MGO generate oxidative stress, our results indicate that ROS, including superoxide anion, interfere with the NO-pathway, in corpora cavernosa, or the EDHF-pathway, in mesenteric arteries, leading to impaired Ach- and EFS-relaxations. Although not addressed in this study, ROS interference with the NO-pathway involves either decreased NO synthesis or increased NO degradation. Increased oxidative stress leads to the oxidation of tetrahydrobiopterin (BH_4_), a cofactor that tightly regulates NO production, resulting in the uncoupling of endothelial NO synthase (eNOS) [32, 33]. In the presence of reduced concentrations of BH_4_, eNOS transfers electrons to molecular oxygen instead of l-arginine to produce superoxide rather than NO [32, 33]. In addition superoxide itself can react and consume NO, forming peroxynitrite and thus enhancing oxidative stress. Hence, NO-mediated relaxations are further impaired. On the contrary, less is known about the interference of oxidative stress with the EDHF-pathway. Impaired EDHF-responses due to increased oxidative stress does not result from altered K^+^ channel function [15]. ROS-induced altered interaction between cAMP and gap junctions communication might represent another explanation [15].

### Resveratrol and quercetin in diabetic conditions

Numerous health benefits have been ascribed to the red wine polyphenols resveratrol and quercetin [16]. Even in diabetic rats these polyphenols restore impaired endothelium-dependent relaxations of aorta [34–36] and improve erectile function [37–40]. In addition, studies have reported a direct anti-diabetic effect of resveratrol and quercetin as they were able to lower blood glucose levels in diabetic rats and mice [41, 42]. In this study resveratrol and quercetin administration prevented glucose/MGO-induced damage to endothelium- and neurogenic-induced relaxations in corpora cavernosa. This is in line with Murat et al. [43] who found that resveratrol protects and restores endothelium-dependent relaxation in hypercholesterolemic rabbit corpus cavernosum. On the contrary, in mesenteric arteries no protective effect of resveratrol or quercetin was found. Different explanations could clarify this tissue specific effect of both polyphenols. First, studies pointed out that resveratrol and quercetin stimulate endothelial NO production [16, 17, 19]. As endothelial- and neurogenic relaxant responses in corpora cavernosa are NO-mediated, enhanced endothelial or neuronal NO synthesis by the polyphenols could clarify the improved Ach-relaxations. In contrast this could not explain the inability of resveratrol and quercetin to protect mesenteric arteries from diabetic-damage to Ach-responses. We have shown that Ach-induced relaxations in mesenteric arteries are EDHF mediated. Nonetheless, one could expect that if resveratrol and quercetin enhance endothelial NO production this could compensate for decreases in EDHF-responses, resulting in improved Ach-relaxations. However this was not the case. Moreover in a previous study [20] we found that resveratrol relaxes mouse corpora cavernosa independent of the endothelium or NO. In addition we also demonstrated that neither resveratrol nor quercetin stimulated Ach-relaxations of mouse corpora cavernosa as such [20]. Therefore improved NO production cannot explain why the protective effect of resveratrol and quercetin against glucose/MGO-induced damage is limited to the corpora cavernosa.

Recently it was suggested that K^+^ channel activation could also be involved in resveratrol’s positive effects on the cardiovascular system [44], including in resveratrol’s relaxant effect on corpora cavernosa [45]. In our study there is no evidence to strengthen this idea, as we previously found that resveratrol relaxes mouse corpora cavernosa independent of K^+^ channel activation [20]. Furthermore this could not explain the protective effect of quercetin on EFS- and Ach-relaxations in corpora cavernosa, since quercetin was not able to relax mouse corpora cavernosa.

Another explanation for the positive effects of resveratrol and quercetin in corpora cavernosa and not in mesenteric arteries, could be found in the different amount of oxidative stress that is induced by the glucose/MGO treatment. Since in mesenteric arteries Ach-relaxations were almost completely abolished by HG and MGO, it is possible that the degree of generated oxidative stress is too high for resveratrol or quercetin to cope with. It is for instance suggested that the protective effects of resveratrol against oxidative injury are attributed to up-regulations of the endogenous cellular antioxidant system rather than to its direct ROS scavenging activity, as it is known that the direct antioxidant effect of resveratrol is rather low [16]. However both polyphenols are as efficacious as known antioxidants to attenuate diabetic-induced oxidative endothelial damage. In addition, in diabetic-mimicking conditions resveratrol has a direct relaxant effect as in the present study resveratrol relaxed corpora cavernosa concentration-dependently in presence of glucose/MGO. Taken together, these results thus suggest a potential therapeutic role for both polyphenols, but especially for resveratrol, in the treatment of diabetes- and oxidative stress-associated ED.

### Limitations

As this study represents a strictly pharmacological in vitro approach, it is hard to extrapolate our results to in vivo circumstances. Although some studies already pointed out that polyphenols in vivo have positive effects on vascular [16] or erectile function [37, 39, 46], further research in this field is definitely required. Furthermore, in our study only the acute effect of glucose/MGO and resveratrol and quercetin administration could be evaluated. Further research will have to confirm whether a similar positive effect on erectile function can be obtained after chronic treatment.

## Conclusions

This study demonstrates that HG in combination with MGO impairs endothelium- and neurogenic-mediated relaxations in mouse corpora cavernosa and mesenteric arteries which is prevented by antioxidants. In arteries, these diabetic conditions primarily affect the EDHF-pathway, while in corpora cavernosa HG and MGO interfere with NO release. Furthermore it was shown that resveratrol and quercetin are effective in preventing HG and MGO deficits of endothelium- and neurogenic-mediated relaxations of mouse corpora cavernosa. Therefore our study further strengthens the strategy of using polyphenols, especially resveratrol, as possible alternative option in the treatment of diabetic ED.

## Abbreviations

AA: ascorbic acid
Ach: acetylcholine
BH_4_: tetrahydrobiopterin
DMSO: dimethylsulfoxide
ED: erectile dysfunction
EDHF: endothelium derived hyperpolarizing factor
EFS: electrical field stimulation
eNOS: endothelial nitric oxide synthase
HG: high glucose
Indo: indomethacin
KRB: Krebs–Ringer bicarbonate
L-NAME: Nω-nitro-l-arginine methyl ester hydrochloride
MGO: methylglyoxal
NHE1: Na^+^/H^+^ exchanger
NO: nitric oxide
Phe: phenylephrine
Quer: quercetin
Res: resveratrol
ROS: reactive oxygen species
SNP: sodium nitroprusside
Temp: tempol
